# Sleep quality and anxiety symptoms in Egyptian children and adolescents during COVID-19 pandemic lockdown

**DOI:** 10.1186/s42269-021-00590-2

**Published:** 2021-07-23

**Authors:** Amira Sayed El Refay, Shaimaa A. Hashem, Hend H. Mostafa, Iman H. Kamel, Lobna S. Sherif

**Affiliations:** Medical Research Division, Department of Child Health, National Research Centre, El-Bohouth Street, P.O. 12622, Dokki, Cairo, Egypt

**Keywords:** COVID-19, Children and adolescent, Lockdown, Sleep quality, Anxiety

## Abstract

**Background:**

Coronavirus Disease Pandemic 2019 has a pervasive effect on all health aspects include psychological and mental health. This study aimed to assess the hidden stressful impact of COVID-19 pandemic on Egyptian children and adolescents’ lifestyles 2 months after lockdown in Egypt by detecting symptoms of anxiety and sleep disorders. Online questionnaire was used by snowball sampling approach 2 months after lockdown targeting children and adolescents.

**Results:**

The overall mean Sleep Disturbance Scale for Children score (SDSC) in participated groups was 44.6 ± 11.72. Of 765 participants 502 (65.6%) showed the symptoms suggestive of sleep disorder. Disorders of initiating and maintaining sleep were the most common among participants as 168 (33.4%) of them were suffering from it while 79 (15.7%) children were suffering from excessive somnolence. Linear stepwise regression revealed that anxiety score, understanding safety measures, and following strict quarantine measures significantly predicted SDSC (*p* = 0.001, 0.009, 0.046). Significant positive correlations were found between SDSC and extra screen usage, understanding safety and quarantine measures, anxiety signs, and change in child lifestyle with (*p* = 0.029, 0.010, 0.001 and 0.001) sequentially. Significant positive correlation was found between family income affection, SDSC, and anxiety with *p* value (00.001, 00.4).

**Conclusion:**

Child deprived of his or her normal lifestyle is vulnerable to develop anxiety symptoms and sleep disturbances. Low income, extra screen time, and restricted quarantine measures are all contributing factors that influence children and adolescent’s mental health.

## Background

The Coronavirus Disease Pandemic 2019 poses an acute influence on all aspects of children and adolescent health including physical, psychological, and mental health (Holmes et al. 2020)

In face of the current pandemic, community control includes measures that range from expanding social distancing to wide community quarantine were applied (Wilder-Smith et al. 2020). Almost all over the world, academic, sports, and social activities have been banned in response to recommendations of social distancing (Villela et al. 2020).

Egypt announced the first confirmed case on late February and immediately the government and the ministry of health created a COVID-19 management team who imposed a sequence of precautionary measures which applied consecutively as schools and universities closure, closure of community activities and finally general lockdown (F 2020).

Although the disease tends to be mild in children, psychological considerations permit us to predict that the pandemic will have a high risk of long-term pediatric psychiatric sequelae (Mastnak 2020).

This study aimed to assess the impact of COVID-19 pandemic on Egyptian children and adolescents’ lifestyles 2 months after lockdown in Egypt by detecting symptoms of anxiety and assess sleep quality and correlate these results with sudden change in their life activities.

## Methods

A 37-item online questionnaire was designed via google form and distributed by snowball method to the parents or caregivers by different ways of social media in which they can respond to questions on behalf of the child. We started on the May 12, 2020, as by this date, 2 whole months of unusual precautions as school closure, community activity limitations, social distancing and finally curfew and quarantine measures were applied. The form started with written electronic informed consent form which explained the aim of the study, the privacy policy, time consumed by the participant, and the benefits risk issues. The form was designed as to end automatically in case of refuse.

For the purpose of validation, the questions were translated into Arabic then the Arabic version in turn was translated into English and the two versions were compared to ensure that it gives the same meaning. A pilot questionnaire in both Arabic and English was distributed first to 15 friends before the full survey to assess the integrity of questions, identify missing response categories, and evaluate any difficulties, and to ensure that the bot version end up with the same score.

- The questionnaire consisted of demographic data as age, gender, residence, education level, and information about the effect of COVID-19 pandemic on the parents’ job and family income. Quarantine measures followed by the family, the symptoms suggestive of anxiety according to CDC criteria were used, and the items were given a score from 1 to 5 for each point for the statistical analysis. Then each participant score was calculated individually to identify the severity of symptoms. Questions about anxiety symptoms included: being very afraid when away from parents, having extreme fear about a specific thing or situation, being very afraid of school and other places where there are people, being very worried about the future and about bad things happening, having repeated episodes of sudden, unexpected fear that come with symptoms like heart pounding, having trouble breathing, or feeling dizzy, shaky, or sweaty and change in appetite (CDC 2020) and *Sleeping quality assessment* using Sleep Disturbance Scale for Children (SDSC) (Bruni et al. 1996) which designed both to evaluate specific sleep disorders in children, and to provide an overall measure of sleep disturbance suitable for use in clinical screening and research. The scale contains 26 items which cover common sleep difficulties affecting children: initiating and maintaining sleep, disorders of arousal, sleep–wake transition disorders and disorders of excessive somnolence. Caregivers use a five-point Likert-type scale to indicate how frequently certain behaviors are exhibited by their children as never, twice per week, 3–4 times per week or daily. An overall score was calculated for each child the cutoff point was 39 scores more than 70 considered as acute sleep disorder.

The sleep quality questions included details of duration of sleep classified by number of hours, if the child arouse from sleep frequently and how many times, if the child needs help from his parent to initiate sleep or main (by asking one of his parent to stay beside her/him during sleep), and these questions were classified into six domains, each domain represents a type of sleep disorder. First, a total score was calculated to evaluate if the child suffers of sleep disorder or not. Then score of each domain was calculated individually to identify the type of sleep disorder.

The study was approved by the Medical Research Ethics Committee (MERC) of The National Research Centre Registration No. is 20092.

Validation of the translation.

### Statistical methods

(SPSS) version 26 (SSPS Inc., Pennsylvania, USA) was used for analysis. Chi-square test was used for comparison, and Spearman’s test correlation analysis was used to assess the relation between two ordinal parameters in the same group. All tests were two-tailed, with a significance level of *p* ≤ 0.05 and highly significance at *p* ≤ 0.01. Linear stepwise regression analysis was used to detect the predictors of the anxiety score and SDSC.

## Results

A total 765 response were received from children and adolescents aged 4–16 years, and the demographic data are presented in Table 1. The majority of the participants were between 6 and 12 years 440 children (57.5%). Of all the participants, 408 children (53.3%) were males and 357 participants (46.7%) were females. More than half of the participants 407 (53.2%) were enrolled in primary schools. An effect on family income was reported in most of participants as a slight effect in 348 children’s families (45.5%) or as a severe effect in 110 participants’ families (27.0%). Most of the participants resided in Greater Cairo including (Giza–Cairo–Qalyubia) (Table 1).

**Table 1:** Sociodemographic characteristics of participants

	Total no (%)	Males no (%)	Females no (%)	Chi-square	(*p* value)
*Gender*					
	765 (100%)	408 (53.3%)	357 (46.7%)	3.400	0.065
*Age groups*					
Less than 6 years	211 (27.6%)	104 (25.5%)	107 (30.0%)	2.205	0.332
6–12 years	440 (57.5%)	244 (59.8%)	196 (54.9%)		
More than 12 years	114 (14.9%)	60 (14.7%)	54 (15.1%)		
*Education stage*					
Nursery	229 (29.9%)	116 (28.4%)	113 (31.7%)	4.030	0.258
Primary	407 (53.2%)	220 (53.9%)	187 (52.4%)		
Preparatory	83 (10.8%)	51 (12.5%)	32 (9.0%)		
Secondary	46 (6.0%)	21 (5.1%)	25 (7.0%)		
*Effect on family income*					
No effect	299 (29.9%)	117 (28.7%)	112 (31.4%)	54.173	0.255
Slight effect	348 (45.5%)	181 (44.4%)	167 (46.8%)		
Severe effect	188 (24.6%)	110 (27.0%)	78 (21.8%)		

*P*: Sig. (2-tailed), *significant at the 0.05 level, **highly significant at the 0.005 level

After 2 months of pandemic, 505 (66%) of participants reported following restricted quarantine measures. The majorities belonged to the age-group 6–12 years. While the children under 6 years found to be were talking about the COVID-19 pandemic more than the other age-groups (623 children (81.4%)). Additionally, 724 participants (94.6%) spend more screen time (Table 2).

**Table 2:** Impact of lockdown on lifestyle according to age group

Lifestyle parameters	Total no (%)			Age groups	Chi-square	*p* value
	(4–6 years) no (%)	(6–1 years) no (%)	(12–16 years) no (%)			
*Follow restrict quarantine measures*						
Follow strict quarantine measures	505 66.0%	143 67.8%	291 66.1%	71 62.3%	0.142	0.931
Do not follow quarantine measures	260 34.0%	68 32.2%	149 33.9%	43 37.7%		
*Understanding safety measures*						
Yes	647 84.6%	155 73.5%	391 88.9%	101 88.6%	27.600	0.001**
No	118 15.4%	56 26.5%	49 11.1%	13 11.4%		
*Talking about COVID-19*						
Yes	623 81.4%	142 67.3%	384 87.3%	97 85.1%	38.819	< 0.001**
No	142 18.6	69 32.7%	56 12.7%	17 14.9%		
*Follow news of COVID-19*						
Does not care	204 26.7%	105 49.8%	78 17.7%	21 18.4%	166.045	< 0.001**
Social media and internet	81 10.5%	5 2.4%	35 8.0%	41 36.0%		
Mass media	64 8.4%	15 7.1%	45 10.2%	4 3.5%		
Family members	266 34.8%	61 28.9%	175 39.8%	30 26.3%		
More than one	150 19.6%	25 11.8%	107 24.3%	18 15.8%		
*Extra screen time*						
Yes	724 94.6%	191 90.5%	423 96.1%	110 96.5%	6.078	0.048*
No	41 5.4%	20 9.5%	17 3.9%	4 3.5%		

P: Sig. (2-tailed), * significant at the 0.05 level, ** highly significant at the < 0.001**level

Of all the participants, 374 (48%) showed symptoms suggestive of anxiety ranging from only sign up to more than one. Seventy-five participants showed symptoms of social anxiety. Only for statistical analysis, a score was given and calculated for these items (Table 3).

**Table 3:** Anxiety symptoms of participants

Participants having	Total number no (%)	Males no (%)	Females no (%)
Anxiety signs	374 (48%)	205(54.8%)	169 (45.1%)

### Sleep Disturbance Scale for Children score (SDSC)

The overall mean Sleep Disturbance Scale for Children score (SDSC) in participated groups was 44.6 ± 11.72 suggesting a widespread stressful impact of the lockdown. A total of 502 (65.6%) children showed the symptoms suggestive of sleep disorder (Table 4).

**Table 4:** Sleep disorder scale total score and sleep disorder types in participants

	Total score Mean ± SD	Male Mean ± SD	Female Mean ± SD	*t*	*p*
SDSC (total score)	44.6 ± 11.72	44 ± 11.477	45.35 ± 12.039	− 1.600	0.110

Sleep disorder	Number	%
Total	502	65.6
Disorder of initiating and maintaining sleep	168	33.4
Disorder of arousal	35	6.9
Sleep hyperhidrosis	16	3.1
Sleep–wake transition disorder	17	3.3
Excessive somnolence	79	15.7
Nonspecific	98	19.5
Combined problems	28	5.5

There was no significant difference in mean scores between males and females 44 versus 45.35, respectively. Overall, only 25 (3.2%) of participants had an (SDSC) over 70 which indicate acute severe sleep disorder (Table 2). Disorder of initiating and maintain sleep was the most common among participated children as 168 (33.4%) of the participated children were suffering from it, while 79 (15.7%) children were suffering from excessive somnolence (Table 4).

By applying linear stepwise regression, it suggested that anxiety score, understanding safety measures, and following strict quarantine measures significantly predicted SDSC (*p* = 0.001, 0.009, 0.046) sequentially (Tables 5, 6).

**Table 5:** Predicting variables of anxiety score by linear stepwise regression analysis

Variable	*B*	SE	Beta	*t*	*p* value
(Constant)	− 0.178	0.173		− 1.029	0.304
Sleep Disorder Score	0.018	0.003	0.209	5.353	< 0.001**
Following news of COVID-19	0.098	0026	0.148	3.787	0.001

Dependent Variable: anxiety score, *p* < 0.001 is highly significant

**Table 6:** Predicting variables of SDSC by linear stepwise regression analysis

Variable	*B*	SE	Beta	*t*	*p* value
(Constant)	41.425	1.967		21.061	< 0.001
Anxiety symptoms score	2.415	0.460	0.203	5.254	< 0.001
Understanding safety measures	3.352	1.282	0.101	2.614	0.009
Follow strict quar. measures	− 1.891	0.945	− 0.078	− 2.000	0.046

^a^Dependent variable: sleep disorder score

Significant positive correlations were found between SDSC and extra screen usage, understanding safety and quarantine measures, anxiety signs, and change in child lifestyle with (*p* = 0.029, 0.010, 0.001 and 0.001) sequentially (Table 7).

**Table 7:** Correlation between sleep disorder score and other lifestyle parameters

	Age	Extra screen time	Understand safety measures	Lifestyle changing	Anxiety signs	talking about COVID-19	Follow COVID-19 news
*SDSC*							
*R*	− 0.032	0.079*	0.093*	0.144*	0.257*	− 0.050	0.039
*P*	0.379	0.029	0.010	0.001	0.001	0.163	0.282

*P*: Sig. (2-tailed), **correlation is highly significant at the 0.005 level, *correlation is significant at the < 0.001**level

*R*: Spearman’s rho correlation coefficient

Significant positive correlation was found between family income affection and sleep disorder score and anxiety with *p* value (00.01, 00.4) consecutively (Table 8).

**Table 8:** Correlation between anxiety, sleep disorder score and change in family income

	Sleep disorder score	Anxiety symptoms score
*Change in family income changing*		
*R*	0.232**	0.115**
*P*	0.001	0.004

*P*: Sig. (2-tailed), *correlation is significant at the 0.05 level, **Correlation is significant at the 0.005 level

*R*: Spearman's rho correlation coefficient

## Discussion

Pandemics usually are not only serious public health concern, rather these generate disastrous social, economic, and political crises in the diseased countries (Chakraborty and Maity 2020).

WHO recommended social distancing and restriction of mass gathering in order to reduce the general risk of transmission of COVID-19 (Chakraborty and Maity 2020). As a result, people around the globe have been asked for self-isolation and refrain from social gathering for months. Social isolation is related to poor mental and may increase the probability of mental disorders (Allocati et al. 2016).

In the current study, we used the criteria suggestive anxiety by CDC to detect the presence of anxiety symptoms. We reported that 374 (48%) participants had one anxiety symptom or more. This high percentage could be explained that in the current study we targeted children and adolescent who considered as a vulnerable group to stressful events, moreover we conducted our study after 2 months of lockdown which could explain the remarkable effect.

Researchers investigated psychological responses during the initial stage of the COVID-19 lockdown. They found that 53.8% of respondents valued the psychological impact of the outbreak as moderate or severe, 16.5% described moderate to severe depressive symptoms, and 28.8% reported moderate to severe anxiety symptoms (Killgore et al. 2020). Another general survey of more than 50,000 people in China through the COVID-19 course revealed that about 35% of the respondents suffered psychological distress (Kessler et al. 2009).

Investigators declared that the ongoing COVID-19 outbreaks has led to an unprecedented public health crisis worldwide. The children and young adolescent were suffering a sudden withdrawal from social activities, disruption normal lifestyle by closing of schools, parks, and playgrounds which in turn can promote stress, confusion, and anxiety. Both children and adolescents are likely to become more demanding, needing to cope up with all these abrupt fluctuations (Wang et al. 2020).

Anxiety disorders are considered common variant of psychological illness, with prevalence of 6–12% and commonly start at childhood and adolescence. It can be present with many signs as variation in appetite, disturbance in sleep, sensitivity, feeling tense, crying without rational cause and negative thoughts (Dubey et al. 2020).

In our study, 75 (9.8%) participants showed symptoms suggestive social anxiety being more adherent to parents in social situations, avoiding gathering places and irritability in case of gathering. This can be explained by several factors as the traumatic events surrounding COVID-19 pandemic (Dubey et al. 2020; Weiner et al. 2015) This is in addition to how the parents reacted to the pandemic including restricted adherence to the quarantine measures, forced changing in the child routine and financial loss (Jones 2020).

The present study reported that majority of children usually express how they think about COVID by talking with their parents. Hence the impact of age stage arises as usually parents are the main support existing to young children after disasters (Zendle and Bowden-Jones 2019). The mechanism underlying the relationship between child and parents’ outcome is unclear, but parents usually serve as role models for coping (Li et al. 2020).

This is in favor to a study that showed a significant correlation between parents’ and children’s concerns concerning H_1_N_1_ flu pandemic of 2009. It also reported extraordinarily positive correlations between children’s fear effects about the disease and H_1_N_1_-associated threat information gained either from media or their families (Pfefferbaum and North 2008).

By analysis of our data, we found that the majority of our participants follows restrict quarantine measures 505 (66%) which translated to less family or friends gathering, less group activity, while 70.1% of participants had experienced financial stress by either slight effect 348 (45.5%) or severe effect 188 (24.6%) on family income.

In our study, a significant positive correlation was found between decrease in family income and anxiety symptoms. These results were in favor to a study conducted on New Orleans households with children experienced the Hurricane Katrina. Poor income families exposed to several negative life events linked to their poverty before and after the event, they experienced a range of negative outcomes including social isolation, physical and emotional health problems, and financial shortage. In the same way, children at this time of COVID-19 may develop post-traumatic phobia after discovering risk information and other annoying facts through media, including social media or by losing one of the family members (Masten and Narayan 2012).

This is in favor to our results as linear regression shows that following the news of COVID-19 is a proxy of being more prone to anxiety symptoms.

Taken together, family reaction to the situation, children’s personalities, social and income changes, children’s perceptions of the lack or balance between their life before the pandemic and after it and media effect serve as a substitution with other many factor as age, gender, and quality of sleep for the psychological impact of the pandemic. We can expect positive and negative symptoms to emerge.

Sleep quality is an indicator for health; stressed children may suffer many sleep problems depending many factors as social and family factors, his or her awareness about the problem, the extent of influence on the quality of life, more utilization of social media, acceleration of screen time using and excessive online gaming enhanced by COVID pandemic (King et al. 2020). Sleep disorders are associated with neurocognitive and psychosocial impairments as well as an increase in caregiver burden (La Greca et al. 2002).

In our study, the overall mean Sleep Disturbance Scale for Children score (SDSC) in participated groups was 44.6 ± 11.72 suggesting a widespread stressful impact of the lockdown.

A research group investigated a relation between social status evaluated by the Personal Social Capital Scale 16 (PSCI-16) and sleep quality in persons who were self-isolated during the COVID-19 outbreak (Wilder-Smith et al. 2020). The authors found that anxiety was associated with stress and reduced sleep quality, and the combination of anxiety and stress reduced the positive effects of social investment on sleep quality. The authors noted that “anxiety and stress of isolated individuals were at elevated levels, while the sleep quality was low.”

Children may suffer from problems initiating or maintaining sleep, physiological problems such as obstructive sleep apnea abnormal or disruptive behaviors during sleep such as sleepwalking, and daytime symptoms such as excessive somnolence (La Greca et al. 2002).

In our study, the disorder of initiating and maintaining sleep was the most common among participant children as 168 (33.4%) of the participated children were suffering from it, while 79 (15.7%) children were suffering from excessive somnolence.

This changes was explained by experts according to the stage of pandemic as the acute phase of the pandemic changes like lockdowns and school closures can activate acute stress reactions and somnolence issues and children can exhibit insomnia and disruptive behavior as they attempt to cope with the strain (Hawkins 2009).

This is followed by the subacute phase. Our lives need to adapt to changed social circumstances, and children could suffer post-traumatic stress disorders that negatively affect developmental processes, personal growth, and cognitive factors (Ahmad and Murad 2020).

Next comes the post-traumatic phase, depending on children’s flexibility and, or, susceptibility to stress, this could initiate self-protective behaviors and personality features, as well as mental health issues such as post-traumatic stress disorders or depression (Moturi and Avis 2010).

In our study, sleep disorders were one of the important findings as 502 (65.6%) children showed the symptoms suggestive of sleep disorder. Moreover, 25 (3.2%) of participants had an (SDSC) over 70 which indicate acute sever sleep disorder which can be explained by the sudden change in lifestyle. Sleep disorders can be associated with many factors; the awareness of the child to the problem as in our results by applying linear regression, we found that children who understand the quarantine measures and who follows the news of COVID pandemic were more prone to sleep disorders. Moreover, a positive correlation was found between sleep disorders and another lifestyle parameter.

Overall screen time utilization was increased in most of our participant either as TV or mobile using or online gaming leading to more disruption of children mental well-being which was translated to nightmares or inadequate sleep quality.

Screen time known to be associated with decrease total sleep period, prolonged initiation of sleep and delayed bedtime. That was supported by many studies that long TV watching, or computers usage time was related to shorter sleep period or delayed bedtime (Zendle and Bowden-Jones 2019; King et al. 2020).

Also, there was a positive correlation between financial instability and the presence of sleep disorders as abrupt deprivation of the child normal activity, or the standard economic level has a great impact on his psychological health.

## Conclusion

Child deprived from normal lifestyle, screen time overuse, awareness of the pandemic and finally family financial instability during lockdowns. All these factors can present with anxiety symptoms and/or sleep disorder which potentially can trigger disruptive and even prolonged adverse mental and cognitive sequalae in children.

Finally, the hidden psychological long-term impact of COVID-19 pandemic should be taken into consideration. Alternative social or activity should be implied to fill the gap of free time induced by the lockdown. A large-scale study will help to assess the real impact of the problem rather than a small, fragmented research.

Families together with schools and community serving authorities should enhance alternative activities to be used by children and young adolescents.

## Limitation of the study

We used the Sleep Disturbance Scale for Children score (SDSC) for screening for sleep disturbance although there were more advanced ways for diagnosis, but it is suitable for research reasons particularly at the quarantine period.

## Data Availability

The author declares the availability of original data by request from Child health department NRC Egypt. Authors may be contacted at Amirasayed.ak@gmail.com.

## Abbreviations

SDSC: Sleep Disturbance Scale for Children score
COVID-19: Coronavirus disease 2019
MERC: Medical Research Ethics Committee
PSCI-16: Personal Social Capital Scale 16
